# Body mass index modified the effectiveness of low dose aspirin treatment on frozen-thawed embryo transfer outcome: a propensity score-matched study

**DOI:** 10.3389/fendo.2024.1365467

**Published:** 2024-04-19

**Authors:** Kaijie Chen, Jiali Cai, Jie Tong, Lanlan Liu, Zhenfang Liu, Jinhua Chen, Xiaolian Yang, Chao Yang, Jie Geng, Caihui Ma, Jianzhi Ren, Xiaoming Jiang

**Affiliations:** ^1^ Reproductive Medicine Center, The Affiliated Chenggong Hospital of Xiamen University, Xiamen, Fujian, China; ^2^ School of Medicine, Xiamen University, Xiamen, Fujian, China

**Keywords:** aspirin, BMI, low body weight, IVF, live birth rate

## Abstract

**Background:**

Low-dose aspirin is one of the widely used adjuvants in assisted reproductive technologies with the hope of improving the live birth rate. However, the studies regarding its effects are conflicting. The study aimed to investigate the association between aspirin administration and live birth following frozen-thawed embryo transfer (FET) in patients with different body mass index (BMI).

**Methods:**

A retrospective cohort study was performed on 11,993 patients receiving FET treatments. 644 of which received a low-dose aspirin (100 mg/day) during endometrial preparation until 10 weeks after transfer. Propensity score matching was performed to avoid selection biases and potential confounders.

**Results:**

The clinical pregnancy rate and live birth rate were similar before matching (54.4% versus 55.4%, RR: 1.02, 95%CI: 0.95-1.09, and 46.3 versus 47.8, RR: 1.03, 95%CI: 0.95-1.12 respectively). A weak association in favor of aspirin administration was found in the matched cohort (49.5% versus 55.4%, RR: 1.12, 95%CI: 1.01-1.24, and 41.9% versus 47.8%, RR: 1.14, 95%CI: 1.01-1.29 respectively). However, when stratified the patients with WHO BMI criteria, a significant increase in live birth rate associated with aspirin treatment was found only in patients with low BMI (<18.5 kg/m2) in either unmatched (46.4% versus 59.8%, RR:1.29, 95%CI:1.07-1.55) or matched cohort (44% versus 59.8%, RR: 1.36, 95%CI: 1.01-1.83) but not in patients with higher BMI categories. With the interaction analysis, less association between aspirin and live birth appeared in patients with normal BMI (Ratio of OR:0.49, 95%CI: 0.29-0.81) and high BMI (Ratio of OR:0.57, 95%CI: 0.27-1.2) compared with patients with low BMI.

**Conclusion:**

BMI may be considered when evaluating aspirin’s effect in FET cycles.

## Introduction

Assisted reproductive technologies (ART) provide a practical option for infertility treatment, but they are still expensive, complex, and fraught with risk of failure (1). To improve live birth rates, many adjuvants and add-ons have been introduced to ART and infertility treatments, though their effectiveness is largely unknown (2). Among these adjuvants, low-dose aspirin is widely used (2). Aspirin is an irreversible inhibitor of cyclo‐oxygenases (COX) 1 and 2 and exerts its effects as an anti-inflammatory, analgesic, and antipyretic medication. The postulated benefits of aspirin as an adjuvant of ART treatment may include improvement in uterine and ovarian blood flow, and inhibiting thromboxane synthesis via inhibition of platelet cyclo-oxygenase, thus preventing thrombosis in the placental vasculature (3). Thromboembolic history or thrombophilia is also a possible etiology for implantation failure (4). Therefore, aspirin might benefit the ART patients with thrombophilia risks.

However, the studies regarding the effect of aspirin in unselected patients are conflicting. Some previous studies showed aspirin might have no substantial effect on improving pregnancy and live birth rates in women undergoing IVF/ICSI (5) or frozen-thawed embryo transfer (FET) (6, 7). However, results from 13 RCTs indicate that aspirin at a dose of 100mg/day may increase the success rate of IVF/ICSI treatment (8). A meta-analysis by Mourad et al. suggested that there was significant evidence that aspirin for endometrial preparation improved live birth rates (9).

On the other hand, some real-world observations which may include larger sample size and wider patient variability support the role of aspirin in improving live birth rates (10–12). Some inconsistencies may lie in the difference in inclusion criteria, the low quality of evidence, and the limited number of available studies. It suggests that the selection bias or patients` characteristics might play a role.

Body Mass Index (BMI) is an important factor to be considered in ART treatment planning and prognosis evaluation. Extreme BMIs may affect ovarian response to gonadotropin stimulation and embryo implantation (13–17). The effect of Aspirin is also affected by BMI in the field other than infertility treatment. High BMI may impair aspirin responsiveness and affect its effect on thrombosis (18) as well as make aspirin more effective in preventing colorectal adenoma (19). However, it is still not clear whether the role of aspirin in ART treatments is similarly affected by BMI. We hypothesize that BMI may be an effect modifier as it did in thrombosis prevention. The present study aims to investigate the association between aspirin and live birth following FET in a patient cohort of different BMI categories. At the same time, live birth outcomes and the interaction between BMI and aspirin have been evaluated.

## Materials and methods

### Study subjects

A retrospective study was conducted on patients who underwent FET treatments in the Center for Reproductive Medicine of the Affiliated Chenggong Hospital of Xiamen University from January 2013 to December 2020. The ethics committee of Xiamen University Medical School gave the retrospective study Institutional Review Board approval. Informed consent was not necessary because the study was carried out with anonymous records that the ethical committee had approved. The BMI classification criteria complied with the worldwide categories of the World Health Organization (WHO) (20). Exclusion criteria were patients in combination use of other adjuvants (n=2915) or the use of prednisone (n=136). The patients with a history of intrauterine adhesion (n=416) were also excluded due to potential confounding.

### Laboratory protocol and endometrial preparation

IVF and ICSI were performed with the routine protocol in our center (21). Vitrification was used for embryo cryopreservation (22), and embryo thawing was carried out using the corresponding thawing kit.

The embryos were transferred either at the cleavage stage (D3) or blastocyst stage (D5 or D6). For cleavage stage embryos, good quality embryos were defined as Grade 1 and 2 cleavage stage embryos according to the Istanbul consensus (23). For blastocysts, good quality embryos were those with ≧BB Gardener score (24) and poor quality embryos were those with C score for either inner cell mass or trophectoderm. However, CC blastocysts were not considered for ET.

The endometrial preparation methods included the natural cycle (NC), modified natural cycle (mNC), hormone replacement therapy (HRT), and the HRT with gonadotropin-releasing hormone agonist (GnRHa) downregulation. In NC cycles, transvaginal ultrasonography was used to track follicle growth from cycle days 9 to 11. Luteinizing hormone (LH) and estradiol levels were checked every three days once the leading follicle’s diameter reached 14 mm. FET was scheduled for the third day and frozen-thawed blastocyst transfer (FBT) was scheduled for the fifth day after ovulation when a spontaneous LH surge was noticed on the day of ovulation (day 0). In mNC cycles, patients are given 6500 units of hCG on the day of the LH surge and intramuscular progesterone injections of 40 mg/day were also started on the second day. The second day following the LH surge was considered to be ovulation day (day 0) and embryo transfer was scheduled accordingly.

In HRT cycles, HRT was carried out using a daily dose of 6 mg of oral estradiol valerate. When the endometrial thickness reached 7-8 mm (designated as day 0), a progesterone injection (40 mg) was administrated, FET was scheduled for four days and FBT was scheduled for six days later. In GnRHa-HRT cycles, a depot of Triptorelin (3.75mg) was administrated on the first day of the menstrual cycle, and estrogen administration was initiated on the first day of the second menstrual cycle. To prevent the possible harm from the cysts, the endocrine profile and follicle were monitored at the initiation of the down-regulation and estrogen administration.

The endometrial thickness and endometrial pattern, as well as the serum estradiol level, were recorded on the day of progesterone administration or the day of ovulation. Under transabdominal ultrasound guidance, embryo transfer was carried out using a Guardia Access Embryo Transfer catheter (K-JETS-7019-SIVF, Cook, IN, USA). Up to the tenth week of pregnancy, luteal support was maintained.

Aspirin was administrated (Bayer Healthcare Manufacturing S.r.l., NMPN: HJ20160685, specification: 100mg/tablet, 30 tablets/box) to patients during the whole endometrial preparation period of the FET cycle, and the administration is not continued until 10 weeks after transfer. The patient assignment is mainly based on the preferences of the patients and the clinicians.

### Statistical analysis

The mean and standard deviation (SD) are used to characterize the distribution of continuous variables. Categorical variables are quantified using percentages and proportions of the whole. Shapiro-Wilke tests, normality plots, t-tests, or Mann-Whitney U tests compare findings for continuous variables. Categorical variables can either pass the Fisher-Price exact test or the Chi-Square test, depending on the circumstance.

Propensity score (PS) matching can avoid potential confounding variables and selection bias because the two groups in clinical practice were not assigned at random (25). These variables included age, BMI, basal endocrine parameters (FSH, LH, and AFC), parity and gravidity, endometriosis, tubal problems, polycystic ovary syndrome (PCOS), history of intrauterine adhesions separation, oocyte yield, ovarian stimulation protocols, ET orders, endometrial preparation protocols, developmental stage of transferred embryos, the number and quality of embryos transferred, and luteal support. Standard differences (D) were used to evaluate the balance of the distribution of the baseline characteristics between the two groups before and after PS matching. D < 0.1 was used as the threshold to indicate a negligible difference in the mean or prevalence of a covariate between exposure groups (26). The propensity score distribution also confirmed the balance (**Supplementary Figure S1**). Using pre-matching data to account for the confounders above, multivariate generalized estimating equations (GEE) models were carried out to validate the findings (27).

To evaluate the modification of BMI on the effect of aspirin, an interaction term (BMI × aspirin) was introduced to the multivariate models in either matched or unmatched cohorts.

## Results

A total of 11,547 patients were included in the study. Of these, 605 (5.2%) patients received aspirin administration (the aspirin group), and 10,969 (94.8%) patients didn’t receive aspirin administration (the non-aspirin group).

Obvious differences appeared in the diagnosis of polycystic ovary syndrome, oocyte yield, and the number, ET order endometrial preparation protocols, the embryo transfer policy, and the luteal phase support methods before PS matching (**Tables 1**, **2**). Following PS matching, a standardized difference of less than 0.1 indicates a negligible difference in the mean or prevalence of a covariate between the study and control groups (**Tables 1**, **2**). An identical distribution pattern between groups was found following PS-matching (**Supplementary Figure S1**).

**Table 1:** Characteristics of patients before and after PS-matching.

Variable	unmatched		P	Standardized difference	matched		p	Standardized difference
	Non-aspirin	Aspirin			Non-aspirin	Aspirin		
	(N=10969)	N=605)			(N=605)	N=605)		
**Female age, year**	31.3 (4.17)	31.6 (4.33)	0.207	0.0526	31.6 (4.02)	31.6 (4.33)	0.912	-0.0061
**Male age, year**	33.2 (4.81)	33.5 (4.73)	0.117	0.0655	33.6 (4.75)	33.5 (4.73)	0.667	-0.0248
**Female BMI, kg/m^2^**	20.9 (2.17)	21.0 (2.12)	0.19	0.0549	20.9 (2.11)	21.0 (2.12)	0.689	0.023
**Male BMI, kg/m^2^**	23.8 (3.36)	23.8 (3.24)	0.893	-0.0056	23.8 (3.34)	23.8 (3.24)	0.928	-0.0053
**Basal FSH,mIU/ml**	7.11 (10.5)	6.92 (2.00)	0.148	-0.0935	6.75 (1.81)	6.92 (2.00)	0.108	0.0882
**Basal LH, mIU/ml**	5.72 (51.3)	5.32 (3.10)	0.429	-0.1292	5.18 (2.85)	5.32 (3.10)	0.438	0.0429
**AFC**	11.9 (5.51)	12.3 (5.56)	0.124	0.0643	12.4 (5.67)	12.3 (5.56)	0.705	-0.022
**Parity≧1**	1812 (16.5%)	87 (14.4%)	0.185	-0.0214	99 (16.4%)	87 (14.4%)	0.381	-0.0198
Gravidity								
0	5699 (52.0%)	313 (51.7%)	0.991	-0.0022	290 (47.9%)	313 (51.7%)	0.474	0.038
1	2753 (25.1%)	150 (24.8%)		-0.003	170 (28.1%)	150 (24.8%)		-0.0331
2	1469 (13.4%)	82 (13.6%)		0.0016	86 (14.2%)	82 (13.6%)		-0.0066
3	655 (6.0%)	39 (6.4%)		0.0047	44 (7.3%)	39 (6.4%)		-0.0083
>3	393 (3.6%)	21 (3.5%)		-0.0011	15 (2.5%)	21 (3.5%)		0.0099
Diagnosis								
Endometriosis	917 (8.4%)	34 (5.6%)	0.0207	-0.0274	32 (5.3%)	34 (5.6%)	0.899	0.0033
Tubal	7126 (65.0%)	399 (66.0%)	0.652	0.0099	421 (69.6%)	399 (66.0%)	0.196	-0.0364
PCOS	916 (8.4%)	70 (11.6%)	0.00722	0.0322	70 (11.6%)	70 (11.6%)	>0.999	0
**Oocyte yield**	13.2 (6.63)	12.8 (6.37)	0.13	-0.0635	12.8 (6.31)	12.8 (6.37)	0.957	-0.0031
Insemination protocol								
ICSI	2349 (21.4%)	110 (18.2%)	0.0246	-0.0323	96 (15.9%)	110 (18.2%)	0.563	0.0231
IVF	7966 (72.6%)	469 (77.5%)		0.049	482 (79.7%)	469 (77.5%)		-0.0215
RICSI	654 (6.0%)	26 (4.3%)		-0.0166	27 (4.5%)	26 (4.3%)		-0.0017
ET order								
1	3776 (34.4%)	180 (29.8%)	<0.001	-0.0467	171 (28.3%)	180 (29.8%)	0.752	0.0149
2	4979 (45.4%)	263 (43.5%)		-0.0192	255 (42.1%)	263 (43.5%)		0.0132
3	1447 (13.2%)	110 (18.2%)		0.0499	122 (20.2%)	110 (18.2%)		-0.0198
>3	767 (7.0%)	52 (8.6%)		0.016	57 (9.4%)	52 (8.6%)		-0.0083

**Table 2:** Characteristics of FET cycles before and after PS-matching.

Variable	unmatched		P	Standardized difference	matched		p	Standardized difference
	Non-aspirin	Aspirin			Non-aspirin	Aspirin		
	(N=10969)	(N=605)			(N=605)	(N=605)		
Endometrial preparation								
GnRHa+HRT	2158 (19.7%)	201 (33.2%)	<0.001	0.1355	186 (30.7%)	201 (33.2%)	0.636	0.0248
HRT	3333 (30.4%)	255 (42.1%)		0.1176	278 (46.0%)	255 (42.1%)		-0.038
Natural cycle	5193 (47.3%)	136 (22.5%)		-0.2486	132 (21.8%)	136 (22.5%)		0.0066
Modified natural cycle	285 (2.6%)	13 (2.2%)		-0.0045	9(1.5%)	13(2.2%)		0.0066
Developmental stage of transferred embryos								
3	2215 (20.2%)	108 (17.9%)	0.103	-0.0234	104 (17.2%)	108 (17.9%)	0.553	0.0066
5	7513 (68.5%)	420 (69.4%)		0.0093	437 (72.2%)	420 (69.4%)		-0.0281
5 + 6	83 (0.8%)	10 (1.7%)		0.009	11 (1.8%)	10 (1.7%)		-0.0017
6	1155 (10.5%)	67 (11.1%)		0.0054	53 (8.8%)	67 (11.1%)		0.0231
7	3 (0.0%)	0 (0%)		-0.0003	–	–		0
The number and quality of embryos transferred								
G	6728 (61.3%)	389 (64.3%)	0.029	0.0296	389 (64.3%)	389 (64.3%)	0.771	0
GG	2163 (19.7%)	96 (15.9%)		-0.0385	91 (15.0%)	96 (15.9%)		0.0083
GGG	26 (0.2%)	1 (0.2%)		-0.0007	2 (0.3%)	1 (0.2%)		-0.0017
GGP	49 (0.4%)	0 (0%)		-0.0045	–	–		0
GP	590 (5.4%)	24 (4.0%)		-0.0141	25 (4.1%)	24 (4.0%)		-0.0017
GPP	43 (0.4%)	2 (0.3%)		-0.0006	2 (0.3%)	2 (0.3%)		0
P	981 (8.9%)	73 (12.1%)		0.0312	82 (13.6%)	73 (12.1%)		-0.0149
PP	337 (3.1%)	16 (2.6%)		-0.0043	8 (1.3%)	16 (2.6%)		0.0132
PPP	52 (0.5%)	4 (0.7%)		0.0019	6 (1.0%)	4 (0.7%)		-0.0033
Luteal support								
D	3105 (28.3%)	57 (9.4%)	<0.001	-0.1889	59 (9.8%)	57 (9.4%)	0.92	-0.0033
D&Ps	1348 (12.3%)	56 (9.3%)		-0.0303	46 (7.6%)	56 (9.3%)		0.0165
D&Ps&S	45 (0.4%)	1 (0.2%)		-0.0024	3 (0.5%)	1 (0.2%)		-0.0033
D&S	2338 (21.3%)	151 (25.0%)		0.0364	152 (25.1%)	151 (25.0%)		-0.0017
Po	237 (2.2%)	41 (6.8%)		0.0462	44 (7.3%)	41 (6.8%)		-0.005
Po&D	3097 (28.2%)	200 (33.1%)		0.0482	213 (35.2%)	200 (33.1%)		-0.0215
Po&D&Ps	199 (1.8%)	11 (1.8%)		0	7 (1.2%)	11 (1.8%)		0.0066
Po&D&S	129 (1.2%)	14 (2.3%)		0.0114	8 (1.3%)	14 (2.3%)		0.0099
Po&Ps	129 (1.2%)	6 (1.0%)		-0.0018	5 (0.8%)	6 (1.0%)		0.0017
Po&S	1 (0.0%)	0 (0%)		-0.0001				0
Ps	11 (0.1%)	2 (0.3%)		0.0023	2 (0.3%)	2 (0.3%)		0
Ps&S	24 (0.2%)	5 (0.8%)		0.0061	6 (1.0%)	5 (0.8%)		-0.0017
S	99 (0.9%)	7 (1.2%)		0.0025	10 (1.7%)	7 (1.2%)		-0.005
Po&Ps&S	207 (1.9%)	54 (8.9%)		0.0704	50 (8.3%)	54 (8.9%)		0.0066

D, Dedrogesterone; Ps, Progesterone soft gels; S, Selenoxone; Po, Progesterone oil; G, Good quality embryo; P, poor quality embryo.

Whether administration of aspirin or not appeared to not affect the endometrial thickness (p=0.562) and estradiol levels (p=0.731) after PS matching (**Table 3**), the clinical pregnancy rates and live birth rates were similar before matching and after matching (**Table 3**). The adjusted ORs for clinical pregnancy and live birth in the matching pre-medication group were 1.14 (95% CI:0.96-1.36) and 1.17 (95% CI:0.98-1.4), respectively. After matching, they were 1.13 (95% CI:0.88-1.45) and 1.28 (95% CI:1-1.64). The RRs for clinical pregnancy and live birth in the matched aspirin group were 1.05 (95% CI: 0.95,1.17) and 1.14 (95% CI: 1.01,1.29), respectively (**Table 3**).

**Table 3:** Outcomes of FET before and after PS matching.

Variable	unmatched		P-value	matched		P-value
	Non-aspirin	Aspirin		Non-aspirin	Aspirin	
	(N=10969	(N=605)		(N=605)	(N=605)	
^a^Endometrial pattern			0.204			0.559
A	228 (2.1%)	8 (1.3%)		5 (0.8%)	8 (1.3%)	
B	9948 (90.7%)	561 (92.7%)		558 (92.2%)	561 (92.7%)	
C	793 (7.2%)	36 (6.0%)		42 (6.9%)	36 (6.0%)	
^a^Estradiol level,pg/ml	396 (410)	505 (533)	<0.001	517 (560)	505 (533)	0.731
^a^Endometrial thickness, mm	9.01 (1.75)	8.27 (1.70)	<0.001	8.21 (1.61)	8.27 (1.70)	0.562
Clinical Pregnancy						
rate, %	5981 (54.5%)	342 (56.5%)	0.357	325 (53.7%)	342 (56.5%)	0.355
RR (95% CI)	Ref	1.04(0.96,1.11)		Ref	1.05(0.95,1.17)	
adjusted OR (95%)	Ref	1.14(0.96,1.36)		Ref	1.13(0.88,1.45)	
Live birth						
rate, %	5095 (46.4%)	297 (49.1%)	0.22	261 (43.1%)	297 (49.1%)	0.0435
RR (95% CI)	Ref	1.06(0.97,1.15)		Ref	**1.14(1.01,1.29)**	
adjusted OR (95%)	Ref	1.17(0.98,1.4)		Ref	**1.28(1,1.64)**	
Early miscarriage						
rate, %	690/5981 (11.5%)	35/342 (10.2%)	0.558	46/325 (14.2%)	35/342 (10.2%)	0.254
RR (95% CI)	Ref	0.89(0.64,1.22)		Ref	0.72(0.48,1.09)	
adjusted OR (95%)	Ref	0.88(0.61,1.28)		Ref	0.76(0.45,1.29)	

a Measured on the day of progesterone administration in HRT cycles or on the day of ovulation in natural cycles.

Bold figures indicate significance at P<0.05 level.

When the patients were stratified according to BMI categories, the association between aspirin administration and live birth was only found in underweight patients regardless the PS matching was carried out or not (**Table 4**). With the interaction analyses, after adjusting for potential confounding factors, the Ratio of OR of live birth comparing aspirin and non-aspirin was significantly lower in the normal-weight patients compared with that in underweight patients, yielding a Ratio of OR of 0.46 (95% CI 0.27-0.77). On the other hand, the Ratio of OR of live births in overweight patients with underweight patients was 0.59 (95% CI 0.27-1.29) (**Table 4**).

**Table 4:** Interaction between aspirin and BMI on live birth rates.

		Live birth	P-value	RR	unadjusted OR	adjusted OR	Ratio of OR
				(95% CI)	(95%CI)	(95%CI)	(95%CI)
Unmatched (N=11547)							
underweight	Non-Aspirin	805/1740 (46.3%)	0.0115	Ref	Ref	Ref	Ref
	Aspirin	48/78 (61.5%)		**1.33(1.11,1.6)**	**1.86(1.17,2.96)**	**2.28(1.38,3.74)**	Ref
Normal weight	Non-Aspirin	3916/8373(46.8%)	0.935	Ref	Ref	Ref	Ref
	Aspirin	225/478 (47.1%)		1.01(0.91,1.11)	1.01(0.84,1.22)	1.08(0.89,1.31)	0.5(0.24, 1.05)
Overweight	Non-Aspirin	374/856 (43.7%)	0.564	Ref	Ref	Ref	Ref
	Aspirin	24/49 (49.0%)		1.12(0.83,1.51)	1.24(0.7,2.2)	1.59(0.82,3.1)	0.73(0.23, 2.35)
Matched (N=1210)							
underweight	Non-Aspirin	37/84 (44.0%)	0.0385	Ref	Ref	Ref	Ref
	Aspirin	48/78 (61.5%)		**1.4(1.04,1.88)**	**2.03(1.09,3.81)**	**2.02(1.06,3.82)**	Ref
Normal weight	Non-Aspirin	209/479 (43.6%)	0.316	Ref	Ref	Ref	Ref
	Aspirin	225/478 (47.1%)		1.08(0.94,1.24)	1.15(0.89,1.48)	1.13(0.86,1.5)	**0.46(0.27, 0.77)**
Overweight	Non-Aspirin	15/42 (35.7%)	0.288	Ref	Ref	Ref	Ref
	Aspirin	24/49 (49.0%)		1.37(0.83,2.25)	1.73(0.74,4.02)	11.85(0.86,164.08)	0.59(0.27, 1.29)

Bold figures indicate significance at P<0.05 level.

**Figure 1:**
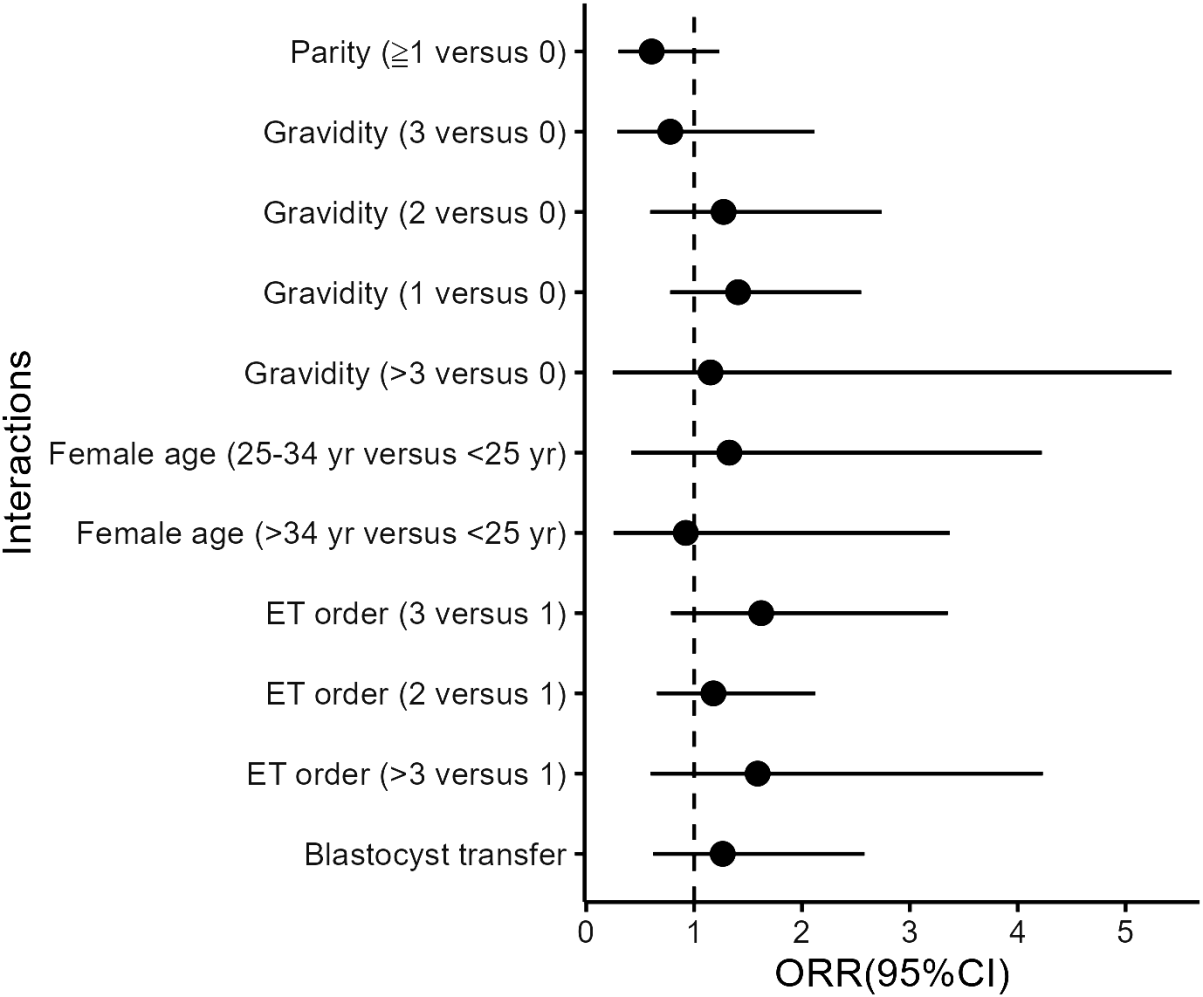
Interactions between aspirin and covariates in the matched cohort. All models were adjusted for age, BMI, basal endocrine parameters (FSH, LH, and AFC), parity and gravidity, endometriosis, tubal problems, polycystic ovary syndrome (PCOS), history of intrauterine adhesions separation, oocyte yield, ovarian stimulation protocols, ET orders, endometrial preparation protocols, developmental stage of transferred embryos, the number and quality of embryos transferred, and luteal support as independent variables.

Besides BMI, we also explored other potential interactions between aspirin use and a series of potential modifiers in the multivariate models, including age, parity, gravidity, previous ET attempts (ET order), and stage of ET (cleavage stage or blastocyst). However, none of these interaction terms reached significance (**Figure 1**).

## Discussion

Our study showed that aspirin administration improved live birth rates in FET patients with low BMI but not in those with normal and high BMI. The results might suggest that the BMI of the patients is a factor to be considered when using aspirin as an adjuvant in FET treatments. The modification of BMI on the effect of aspirin in FET cycles may also partially explain the heterogeneity among previous studies and suggest future evaluations of aspirin effect based on BMI category.

While little evidence was documented regarding the interaction between BMI and aspirin on live births following ART treatment, our study may be supported by a study using aspirin in treating pregnancy loss (27). In the study, Sjaarda et al. discovered that the live birth rate of women with normal BMI and below would increase by 60% when receiving low-dose aspirin treatment, and this effect will be highlighted with the reduction of BMI, however, no obvious effect was seen in women with high BMI, suggesting the effect of aspirin administration might be modified by BMI. Nevertheless, the trial also included folate acid and the scenario was different from the FET treatment.

Several previous studies have suggested a neutral effect of aspirin on embryo transfer outcomes. The range of BMI in these studies suggested a considerable difference from ours. For instance, Motteram et al. have shown that aspirin administration during FET cycles had no positive effect on live birth rates (28). In their study, the average BMI of the FET cycle group was 25.4 ( ± 4.9), which is in the normal to overweight range. The study by Davar et al. came to similar results in a small population with a proportion of high BMI patients (>24.5 kg/m2) of 68.75% (29). Other studies reported that aspirin had no effect on live birth rates in FET cycles and did not provide information about BMI (30, 31). They are based on Spanish and American populations. Considering the lifestyles and distribution of BMI in the general population in Western countries (32–35), the BMI of patients in these studies may be higher than ours.

In a Chinese population, He et al. showed no positive effect on live birth rates with short-term (7 weeks) aspirin at 50 mg per day in women receiving procedural FET (36). While the average BMI of their patients remained higher than ours, the difference in the dosage and duration of aspirin might also contribute to the different conclusions.

For endometrial preparation, aspirin initiation time in the above study (28, 29, 37, 38) is generally consistent with this study, starting from the first day of menstruation. However, most studies use aspirin until 36 weeks if the patient is pregnant or even up to 40 weeks while we stopped the use of aspirin ten weeks after following transfer. Although the time to discontinuation was earlier than in other studies, it may suggest that this time was sufficient to improve reproductive outcomes. On the other hand, aspirin administration in fresh transfer cycles may initiate on the day of stimulation (39), which is supposed to not only affect the endometrium but also the embryos.

As shown in a previous study, the pharmacodynamics of aspirin are weakened by a greater BMI (18). When using elevated TXB2 (Thromboxane B2) levels as a marker of incomplete response to aspirin (>2.2 ng/ml), Maree et al. showed that a significant part of patients (44/131) receiving aspirin 75mg daily may have an inadequate response to treatment. In this work, the patients` weight is associated with inadequate response, as shown in a multiple regression analysis including age and other confounders (40). Another study with healthy subjects suggested that subjects with TXB2 inhibition failure by aspirin have a greater weight and an 80kg subject may have a 20% probability of aspirin response failure (41). Increased body weight or obesity has been listed among the long-lasting sources of variable aspirin response (42). Being obese may lead to several physiological changes, including changes in body composition, regional hepatic blood flow, plasma proteins and/or tissue components, distribution volume, kidney and hepatic clearance rates, as well as the activity of some key enzymes such as CYP450s are also increased in obese patients (42), all these factors might contribute to lower bioavailability of aspirin. On the other hand, obesity is also associated with hyperactive platelets with the presence of pro-thrombotic plasmatic molecules (43). In a large meta-analysis, Rothwell et al. showed that the ability of 75–100 mg aspirin to reduce cardiovascular events decreased with increasing weight and they showed that the loss of effect at larger body size is driven more by weight and height than by BMI, suggesting insufficient systemic bioavailability of aspirin rather than increased platelet activation secondary to obesity (44). Therefore, it might suggest a necessity for weight-based dosing of aspirin considering the bioavailability.

In ART treatment, aspirin is used as an anticoagulant and anti-inflammatory adjuvant, administrated during ovarian stimulation, endometrial preparation, or following embryo transfer. However, evidence regarding the effect of aspirin on the outcomes remained inconsistent, according to several recent meta-analyses analyses that summarized the available lectures (3, 8, 9). In these studies, patients typically receive a daily dose of 80-100 mg aspirin and a dosage <150 mg is considered “low-dose” (3). However, the determination of aspirin dosage seldom considers patients` BMI or body size, although adjusted dosing is commonly used in the practices, such as ovarian stimulation. In most IVF studies, 100 mg daily aspirin was used, which is similar to the dosage used by Petrucci et al. where obesity impaired aspirin responsiveness (18). According to Petrucci et al., the aspirin bioavailability could be rescued by 200 mg aspirin once daily or 85 mg twice daily (18). However, when increasing aspirin dosage was considered, the side effects of aspirin, such as nausea and bleeding should also be taken into consideration.

The effect of aspirin might also relate to the characteristics of lean patients. Being underweight may also play a role in thrombosis (45). In underweight individuals receiving atrial fibrillation anticoagulation, the risks of thromboembolism (RR 1.92, 95% CI:1.28, 2.90) were considerably elevated (46). On the other hand, placenta pathways regulating placental nutrient metabolism and angiogenesis might be associated with maternal pre-pregnancy underweight status (47), suggesting the pregnancy might need more proangiogenic support. As shown in our previous studies and others (48), low BMI patients are associated with poor ART outcomes. Aspirin is supposed to favor pregnancy by its proangiogenic, antithrombotic, and anti-inflammatory effects (49). These effects may partially rescue the suboptimal maternal environment that is associated with underweight and thus improve the outcomes. On the other hand, the previously mentioned weight-dose interaction also exists in lean patients. In comparison with normal-weight or overweight patients, the systemic bioavailability of aspirin might be higher and therefore lead to higher effectiveness as well as greater risks of potential hazard associated with aspirin use (44). To this end, future prospective studies focused on the effect of aspirin on low BMI women are warranted.

The strength of the study includes a larger sample size compared to previous studies and a PS-matching design. The studies also had several limitations. First, the study was retrospective, which may include bias and unmeasured confounders. Second, the study population does not have a clear indication of the use of aspirin. As the risk factors for thrombosis are not routinely examined in our population, they also could not be used as covariates for PS matching. Third, the response to aspirin in the patients is unknown and the antithrombotic effect or other effects of aspirin in the patients are yet not to be confirmed. Finally, the use of aspirin is restricted in FET cycles in our study, it is not clear whether BMI also plays a role in fresh cycles.

## Conclusion

In conclusion, our study showed patients with low BMI are more likely to benefit from using low-dose aspirin in FET cycles than patients with higher BMI. The data may suggest that BMI should be considered when evaluating aspirin’s effect in FET treatment. However, the exact reason for the phenomenon and a causal relationship remains to be elucidated.

## Data availability statement

The original contributions presented in the study are included in the article/**Supplementary Material**. Further inquiries can be directed to the corresponding authors.

## Ethics statement

The studies involving humans were approved by the ethics committee of Xiamen University Medical School. The studies were conducted in accordance with the local legislation and institutional requirements. Written informed consent for participation was not required from the participants or the participants’ legal guardians/next of kin because the studies were carried out with anonymous records that the ethical committee had approved.

## Author contributions

KC: Conceptualization, Data curation, Formal analysis, Resources, Writing – original draft, Writing – review & editing. JCa: Conceptualization, Data curation, Formal analysis, Resources, Writing – original draft, Writing – review & editing, Funding acquisition, Methodology. JT: Writing – original draft, Writing – review & editing. lL: Writing – review & editing. ZL: Writing – review & editing. JCh: Writing – review & editing. XY: Writing – review & editing. CY: Writing – review & editing. JG: Writing – review & editing. CM: Writing – review & editing. JR: Writing – review & editing. XJ: Writing – review & editing, Writing – original draft.
