# Supplementary figures and images for: Body mass index modified the effectiveness of low dose aspirin treatment on frozen-thawed embryo transfer outcome: a propensity score-matched study
Source: Front Endocrinol (Lausanne). 2024 Apr 19;15:1365467. doi: 10.3389/fendo.2024.1365467 (PMC11067524; doi:10.3389/fendo.2024.1365467)

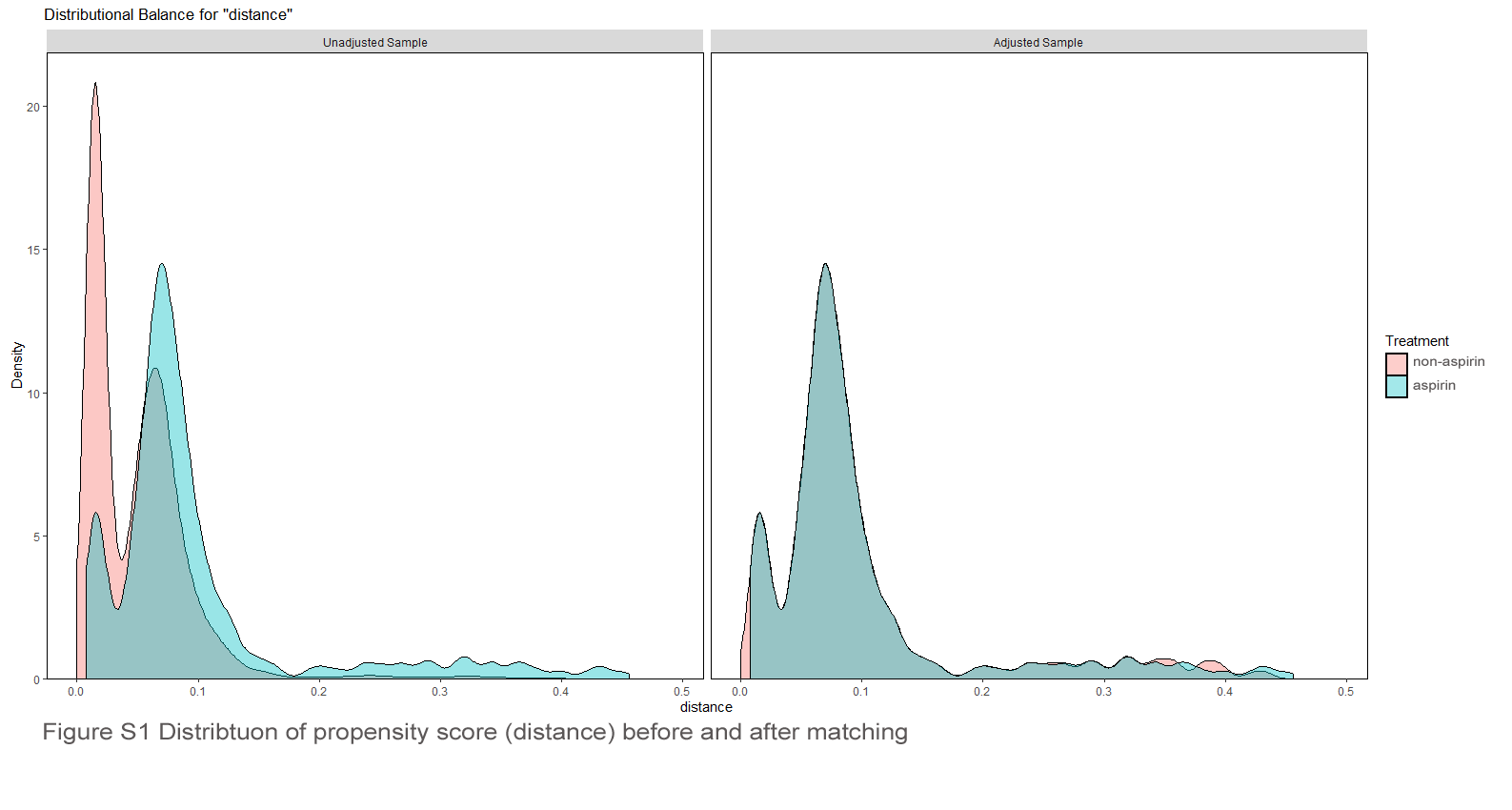

Supplement: Supplementary file 1 [file Image_1.tiff]
